# Targeted drug discovery and development, from molecular signaling to the global market: an educational program at New York University, 5-year metrics

**DOI:** 10.15761/JTS.1000215

**Published:** 2018-02-16

**Authors:** Gloria Lee, Joseph Plaksin, Ravichandran Ramasamy, Gabrielle Gold-von Simson

**Affiliations:** 1 T35 NIDDK Honors Trainee, Clinical and Translational Science Institute, New York University School of Medicine, NY, NY, USA; 2 State University of New York at Downstate College of Medicine, Brooklyn, NY, USA; 3 Department of Medicine, Duke University School of Medicine, Durham, NC, USA; 4 Departments of Medicine, Biochemistry and Molecular Pharmacology, co-PI NIDDK R25, New York University School of Medicine, New York, NY, USA; 5 Department of Pediatrics, PI NIDDK R25, New York University School of Medicine, New York, NY, USA; 6 Clinical Translational Science Institute, New York University School of Medicine, New York, NY, USA

**Keywords:** career, drug development, drug discovery, education, graduate student, translation

## Abstract

Drug discovery and development (DDD) is a collaborative, dynamic process of great interest to researchers, but an area where there is a lack of formal training. The Drug Development Educational Program (DDEP) at New York University was created in 2012 to stimulate an improved, multidisciplinary DDD workforce by educating early stage scientists as well as a variety of other like-minded students. The first course of the program emphasizes post-compounding aspects of DDD; the second course focuses on molecular signaling pathways. In five years, 196 students (candidates for PhD, MD, Master’s degree, and post-doctoral MD/PhD) from different schools (Medicine, Biomedical Sciences, Dentistry, Engineering, Business, and Education) completed the course(s). Pre/post surveys demonstrate knowledge gain across all course topics. 26 students were granted career development awards (73% women, 23% underrepresented minorities). Some graduates of their respective degree-granting/post-doctoral programs embarked on DDD related careers. This program serves as a framework for other academic institutions to develop compatible programs designed to train a more informed DDD workforce.

## Introduction

There is a revived optimism despite many challenges in drug discovery and development (DDD) [1–3]. DDD educational programs can influence growth and development of novel therapies and market transition [4]. Due to escalating complexity and feasibility barriers, there is a significant need for DDD educational programs. The median cost and time to develop a potential molecule from its discovery to approval is 2.6 billion dollars and 36 years, respectively [5,6]. Only 10 to 20% of health-related research projects reach human trials; only 6% of drugs that enter phase I trials reach approval status [7,8]. The new drug approval rate has remained low [9,10]. Therefore, to promote successful translation in DDD, scientists need skills and training [11]. Few DDD training programs integrate aspects on both discovery and development and expose students to opportunities and experts in the field [12].

In September 2012 with funding from the National Institute of Diabetes and Digestive and Kidney Diseases (NIDDK), we began enrollment for the Drug Development Educational Program (DDEP) at New York University (NYU). We aim to teach graduate and postgraduate students the essentials of DDD and inspire these motivated and talented individuals to get more involved in the translation of scientific discoveries and enter the DDD workforce as trained scientists, engineers, business analysts, and entrepreneurs. This improved, informed DDD workforce will be better equipped to address the nation’s biomedical and clinical research needs.

Our educational objectives were met, consistent with reported data from our two-year analysis [13]. Furthermore, we explored *why* students enrolled in each course, *what* they learned, and the course’s relevance to their career goals. We also traced the career paths of students who received DDEP’s career development awards. DDEP aims to teach essential skills and is the only known program of its kind within the Clinical and Translational Science Institutes (CTSIs).

## Materials and methods

### Description of course series

The NYUSOM DDEP was created in 2012 with support from NIDDK. Currently in its sixth year, the DDEP was initially comprised of a two-course series designed to highlight the essential and innovative features of the DDD process. Our goals are: 1) to teach a new generation of multidisciplinary students at the graduate and post-graduate levels about DDD; 2) to potentiate and expedite the translation of therapeutics and other applications, both novel and repurposed; and 3) to bridge the translational gap.

Enrollment is open to all NYU graduate and post-graduate students. The first course of the two-course series is Drug Development in a New Era (DDNE). This course is held in the fall term and focuses on the post-compounding aspects of DDD such as intellectual property, regulatory aspects, and marketing. The spring term course, Molecular Signaling in the Discovery and Development of Therapeutics (MSDDT), expands on molecular signaling pathways as potential sites for drug targets as well as viability testing (Table 1). Each course grants three credits. Classes are two hours in duration, where each begins with a lecture given by an expert in the field followed by an interactive discussion. Students are required to arrive prepared, having completed the assigned reading and coursework.

### DDNE (fall) course

The goal of the DDNE course is to provide an overview of the regulatory, economic, ethical, and business aspects of DDD from the preclinical phase through post-approval marketing and monitoring. Students are exposed to a wide range of topics including protocol planning, safety monitoring, cost and pricing analysis, and intellectual property. Students also learn how basic and clinical sciences, statistical analysis, business management, legal, and marketing departments converge in this interdependent, multidisciplinary process. At the end of the term, students are required to complete a final project that entails submission of a “mini” Investigational New Drug Application (IND) [7].

### MSDDT (spring) course

In the MSDDT course, the principles of discovering and developing therapeutics in the lab as well as essential signaling pathways such as RAS and AKT are explored. Students learn about entity-specific paradigms that can help predict successful DDD trajectories and how to plan target selection via experimental testing. To ensure students are ready for the different scientific aspects of DDD, students are exposed to topics such as developing receptor and pathway networks, prediction models, new technologies, and challenges of designing animal models and clinical trials. Students are required to submit a final paper on research methods or approaches used to design therapy [8].

### Student demographics

Demographic information, training level, and graduate school affiliation was collected from students who enrolled between Fall 2012 and Spring 2017.

### Program evaluation

#### Pre/Post course surveys and analysis

To evaluate students’ self-reported contextual knowledge, each student completed a pre course survey on the first day of the course and a post course survey on the last day of the course. The surveys asked students to rate how well they understood each of the topics/knowledge items. The response options were: (1) nothing, (2) almost nothing, (3) some and (4) a great deal. Responses to all knowledge questions were summed to create a total knowledge score. Each survey also included a question about the relevance of the course in terms of career goals. The choices were: (1) not at all relevant, (2) only a little relevant, (3) somewhat relevant and (4) very relevant. IBM SPSS was used to analyze the differences between the pre and post course surveys. Due to small sample size and non-normally distributed data, Wilcoxon Ranked Sum Test was used for all data analyses.

#### Lecture ratings

On the last day of each course, students received surveys asking them to rate each of the lectures in 4 different domains: content, presentation, relevance, and overall. The options for each response were: (1) poor, (2) lower than expected, (3) satisfactory, (4) above expectations, (5) superior. The response to the question “Was the lecture free of commercial bias?” was also analyzed.

#### Free-response text

To capture more qualitative information, each survey contained free-response questions. In the pre course survey, students were asked, “What are the main things you hope to get out of this course?” They were also asked to explain their rating for the career relevance question. In the post course survey, students were asked, “What are the main take home points you got out of this course?”

IBM SPSS Text Analytics for Surveys was used to analyze the answers to free–response questions. The responses were grouped into categories for query and associated with keywords the students utilized in the response. We manually excluded words that contained components of search terms. Likewise, some responses that were not automatically grouped into the corresponding categories were grouped manually based on content. For the questions “What are the main things you hope to get out of this course” and “What are the main take home points you got out of this course”, Chi-square test was used to compare the differences between the pre and post course surveys.

#### Career development award

Enrolled students were eligible for a career development award. The goal of the award was to provide support for students to pursue activities to advance their careers. Uses included tuition remission, workshops, course/conference attendance, and publication support. Online search and telephone follow-up were used to track recipient’s career trajectories.

## Results

### Student demographics

From September 2012 through June 2017, 139 students completed the DDNE course, and 75 students completed the MSDDT course. A total of 196 students completed at least one course in the DDEP; 18 (9%) students completed both DDNE and MSDDT.

### DDNE (fall) course

**Figure 1:**
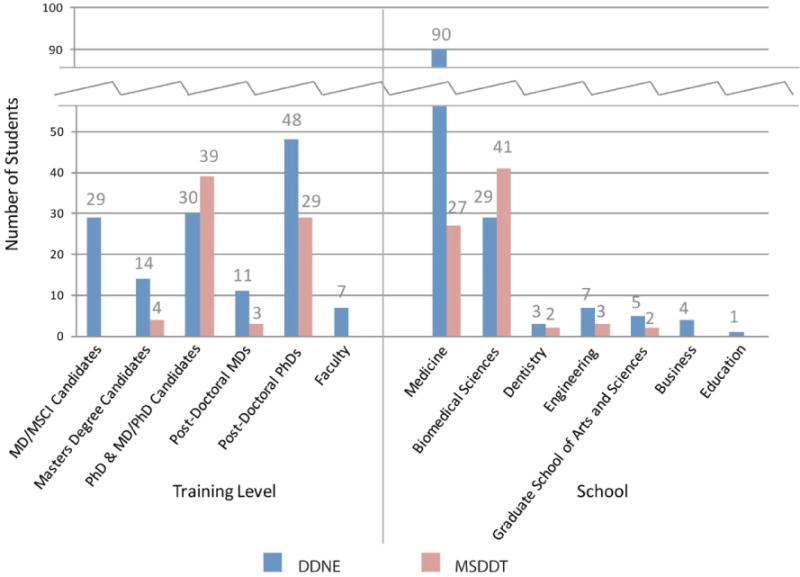
Student demographics This bar graph shows the student demographic data: enrollment numbers, training levels, and affiliated schools for both DDNE and MSDDT courses.

The 139 DDNE students were comprised as such: 48 (35%) postdoctoral PhD, 30 (22%) PhD or MD/PhD candidate, 29 (21%) MD/Masters of Science in Clinical Investigation dual-degree candidate (MD/MSCI), 14 (10%) other Master’s degree candidate, 11 (8%) post-doctoral MD, and 7 (5%) faculty. 90 students (65%) were either enrolled or held positions at NYUSOM, 29 (21%) at NYU Sackler Institute of Graduate Biomedical Sciences, 7 (5%) at NYU Tandon School of Engineering, 5 (4%) at NYU Graduate School of Arts and Sciences (GSAS), 4 (3%) at NYU Stern School of Business, 3 (2%) at NYU College of Dentistry, and 1 (1%) at NYU Steinhardt School of Culture, Education, and Human Development (Figure 1).

### MSDDT (spring) course

The 75 students who completed the MSDDT course were comprised as such: 39 (52%) PhD or MD/PhD candidate, 29 (39%) post-doctoral PhD, 4 (5%) Master degree candidate, and 3 (4%) post-doctoral MD. 41 students (55%) were either enrolled or held positions from NYU Sackler, 27 (36%) from NYUSOM, 3 (4%) from NYU Tandon, 2 (3%) from NYU GSAS, and 2 (3%) from NYU Dentistry (Figure 1).

### Program evaluation

#### DDNE (fall) course

##### Pre/Post course surveys

**Figure 2:**
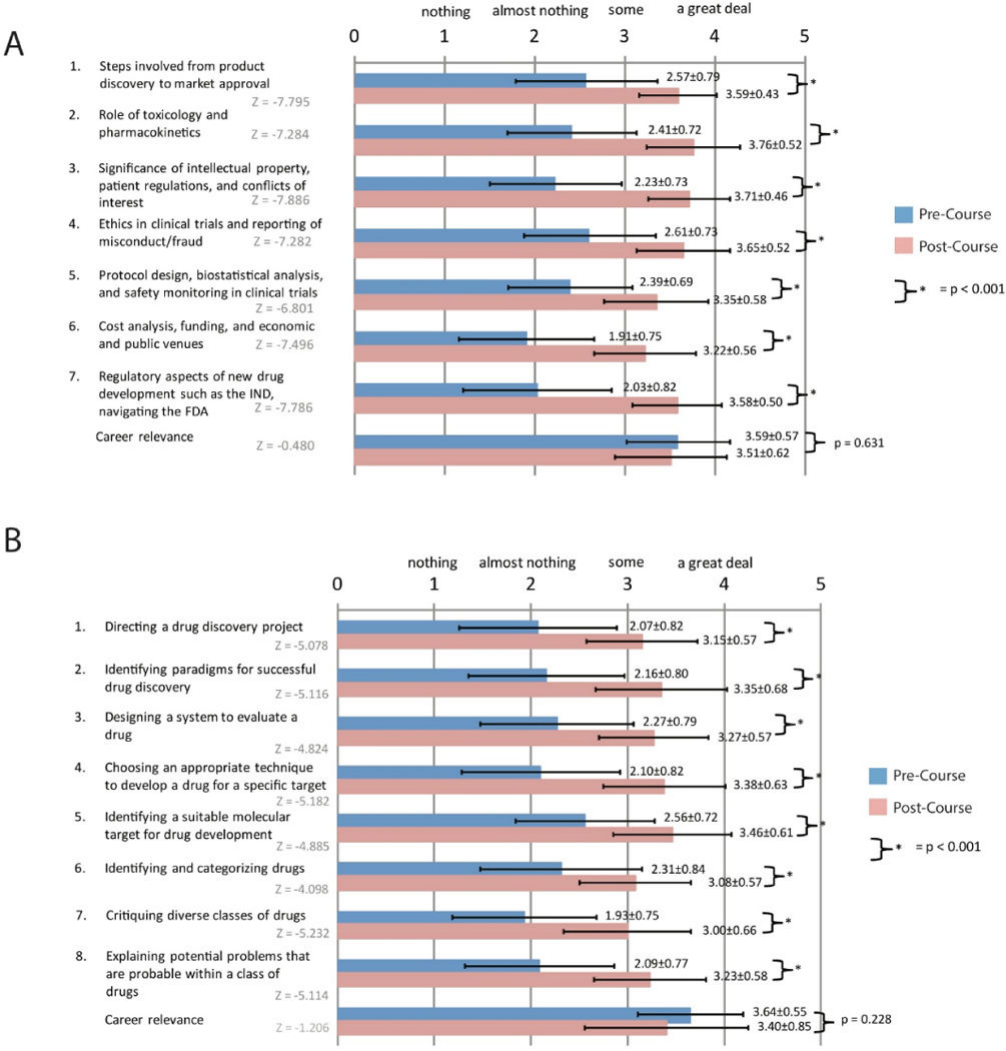
Pre and post course survey results. (A) This figure shows the results of the pre and post course surveys for the DDNE Course. (B) This figure shows the results for the MSDDT Course. The survey choices were (1) nothing, (2) almost nothing, (3) some, and (4) a great deal. The mean ratings and standard deviations are shown. Students rated themselves to be more knowledgeable in all areas after taking both the DDNE and MSDDT courses respectively (p < 0.001). There is no statistical difference in career relevance for either course.

139 students completed DDNE between September 2012 and June 2017. Of those, 127 (91%) completed the pre course survey, 98 (71%) completed a post course survey, and 89 (64%) completed both pre and post course surveys. Responses to all the individual knowledge questions, the total knowledge score, and the career relevance question were not normally distributed (pre course total knowledge score: Kolmogorov-Smirnov p = 0.006 and Shapiro-Wilk p = 0.114; all others: Kolmogorov-Smirnov p ≤ 0.001 and Shapiro-Wilk p ≤ 0.001). Students rated themselves significantly higher in each of the knowledge areas as well as overall knowledge after taking the course. The mean pre course career relevance (3.59 ± 0.57) was not different from the post course career relevance (3.51 ± 0.62) (Z=−0.480; p = 0.631) (Figure 2A, Table 2).

##### Lecture ratings

94 (68%) students completed the lecture ratings (Range 1-5). The mean ratings for each domain were: content 4.17 (±0.80), presentation 4.12 (±0.87), relevance 4.25 (±0.81), and overall 4.18 (±0.81). 95% (1062 of 1116 responses to all lectures) reported that lectures were free of commercial bias.

##### Free response text

113 (89%) of the 127 students who completed the pre course survey explained their career relevance ratings. 66 (58% of the 113 responses with explanations) indicated interest in working in research and DDD related careers. 40 (35%) indicated interest in a career outside academia (Table 3).

In the pre course survey, 94 (74% of the 127 responses) students responded to the question “what they hope to get out of the class.” 35 (37% of the 94 responses to this question) indicated they were interested in the business and/or marketing aspects of DDD, 14 (15%) were interested in learning about the regulatory aspects, and 1 (1%) indicated interest in intellectual property (Table 4). In the post course survey, 92 (94% of the 98 responses) students answered, “what were the main takeaways of the class.” 31 students (34% of the 92 responses to this question) indicated they learned about the business and marketing aspects of DDD, 20 (22%) said they learned about regulatory aspects, and 13 (14%) learned about intellectual property. In the post course surveys, there is a significant increase (χ^2^ p = 0.034) in the number of responses that include keywords in the intellectual property category (Table 4).

#### MSDDT (spring) course

##### Pre/Post course surveys

75 students completed the MSDDT course over five years, 68 (91%) completed the pre course survey, 52 (69%) completed a post course survey, and 46 (61%) completed both pre and post course surveys. Responses to all the individual knowledge questions, the total knowledge score, and the career relevance question were not normally distributed (pre course total knowledge: Kolmogorov-Smirnov p = 0.004 and Shapiro-Wilk p = 0.005; post course total knowledge: Kolmogorov-Smirnov p = 0.004 and Shapiro-Wilk p = 0.226; all the others: Kolmogorov-Smirnov p ≤ 0.001 and Shapiro-Wilk p ≤ 0.001). As was the case in DDNE, students rated themselves significantly higher in each of the knowledge areas as well as total knowledge score after taking the course. Similarly, there was no difference between the pre and post course career relevance ratings (pre course: 3.64 ± 0.55; post course: 3.40 ± 0.85; Z = −1.206, p = 0.228) (Figure 2B, Table 2).

##### Lecture ratings

51 (68%) students in the MSDDT course completed the lecture ratings (Range 1-5). The mean ratings for each domain were: content 3.96 (±0.85), presentation 3.95 (±0.92), relevance 4.06 (±0.85), and overall 4.01 (±0.88). 98% (489 of 498 responses to all lectures) of responses reported that lectures were free of commercial bias.

##### Free response text

55 (81%) of the 68 students who completed the pre course survey explained their career relevance ratings. A majority of responses (37, 67% of the 55 responses with explanations) indicated interest in working in research and DDD related careers. 17 (31%) students indicated interest in a career outside academia (Table 3).

In the pre course survey, 63 (93% of the 68 responses) responded to the question, “what they hope to get out of the class.” 32 (51% of the 63 responses to this question) indicated they were interested in learning how to discover a drug, 9 (14%) were interested in the business and/or marketing aspects of DDD, 2 (3%) were interested in learning about the regulatory aspects (Table 4). In the post course survey, 47 (90% of the 52 responses) students answered “what were the main takeaways of the class.” 24 (51% of the 47 responses to this question) students indicated they learned how to discover a drug, 9 (19%) learned about the business and marketing aspects of DDD, 3 (6%) learned about regulatory aspects, and 1 (2%) learned about intellectual property (Table 4).

### Career development award

**Figure 3:**
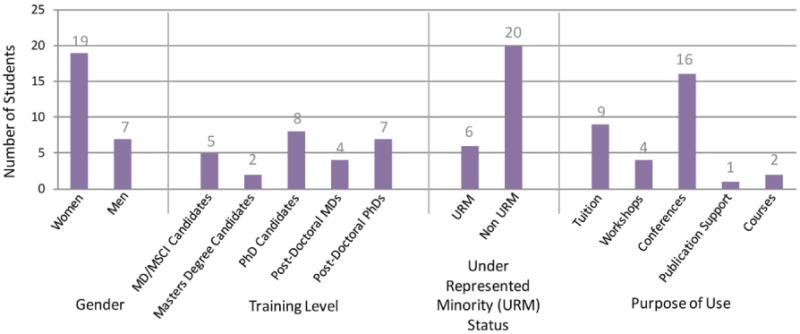
Career development award recipients This bar graph displays the number of career development awardees categorized by gender, training level, underrepresented minority status, and purpose of use. Of note is that some students used career development funds for more than one purpose.

26 students were granted funds for career development activities. A total of $92,250 was awarded. 8 (31%) awardees were PhD candidates, 7 (27%) post-doctoral PhDs, 5 (19%) MD/MSCI candidates, 4 (15%) post-doctoral MDs, and 2 (8%) were Master’s degree candidates. 19 (73%) of the award recipients were women. 6 (23%) of the awardees were under-represented minorities (URM). 16 (62%) of the students used the funds to attend conferences, 9 (35%) for partial tuition remission, 4 (15%) for grant writing workshops, 2 (8%) to enroll in mini-courses, and 1 (4%) for publication support (Figure 3).

### Where are the students who received career development support after graduation from their primary matriculated program/appointment?

The five graduated medical students who received support are all currently enrolled in US residency programs. Two of the eight PhD students are now graduated; one has a position as a post-doctoral trainee and one is a consultant at a healthcare company. Two Master’s degree students graduated; one is a project coordinator at a healthcare company and the other is a clinical trials research coordinator. All four post-doctoral MD students are now assistant professors at academic institutions. One of the seven post-doctoral PhD students is a manager at a pharmaceutical company; the other six continue their post-doctoral training (Table 5).

## Discussion

Our program continues to attract talented and motivated students from a variety of backgrounds at an early career stage. Initially, enrollment included students from Schools of Medicine, Biomedical Sciences, and Arts and Sciences. Currently, the student base has expanded to include the Schools of Dentistry, Engineering, Business, and Education. Moreover, our lecturers have diverse, yet specific, expertise from the various realms of DDD including academia, government/regulatory agencies, industry (large and small), and the business/economic sector. As a result, the lectures and discussions provide a platform for the multidisciplinary students to exchange ideas and share experiences toward a common goal.

The students who received career development awards are arguably our most enthusiastic students. Funds were used most often for conference attendance, which shows that recipients have strong interests in networking and becoming more fluent in their field of interest. Some of these awardees have embarked on careers in competitive medical fields and others are directly involved in DDD. It has been shown that MD/MSCI students apply to more competitive residency programs, and the current institutions and specialties of our MD/MSCI graduate recipients reflect this tendency [9]. Our program is successful in preparing these students to be key players in DDD. In addition, our career development funding encourages URM students. 23% of our recipients are URMs, while only 8.3% of all science, technology, engineering and mathematics PhDs are URMs [10]. Likewise, women have historically been underrepresented in science and industry but 73% of our career development recipients are women [11].

In the first two years, there were 37 and 23 students in DDNE and MSDDT, respectively [6]. After five years, these numbers have increased to 139 and 75, respectively, reflecting increased annual enrollment in both courses without compromising the quality of lectures and discussions. With a larger sample size, we found consistently high lecture ratings in both courses, and students demonstrated learning in all areas queried. The high post course survey knowledge ratings suggest that our course can give students the knowledge, networking abilities, and confidence needed to successfully participate in DDD projects. In addition to gained knowledge, we can ascertain from the free text responses to “what students got out of the course” that students learned about the regulatory, business, economic, marketing, and intellectual property aspects. Furthermore, in regards to the latter, the number of responses that contained the keyword intellectual property increased significantly in the post-course DDNE survey. This is likely due to the course’s highly rated lecture on intellectual property [14–19].

Regarding career relevance, the majority of students (58% DDNE, 67% MSDDT) stated a desire to pursue a research and/or DDD related career. Approximately 1/3 of students indicated interest in working outside academia. Current trends in PhD training programs show that only 12.8% of graduates ultimately end up in academic careers [12]. Additional educational programs such the DDEP are important programs to enable students to gain skills and potentiate the DDD workforce.

The authors acknowledge that since there are no final exams (post-test) in our course series (and we did not administer a pre-test), we do not have a quantitative measurement of knowledge gained. The career trajectories of only those students who received the career development awards were examined.

## Conclusion

Our DDEP program is successful in teaching DDD to students at an early career stage. Despite the fact that our students are at different levels of training and from different schools/fields, the material is appropriate and relevant. By inviting speakers from different areas of DDD and maintaining a discussion-based forum with multidisciplinary students, our program is able to provide collaborative, yet efficient training. We hope to produce highly trained scientists, engineers, educators, business analysts, and other innovators in DDD to further fuel the process. We will continue to expand the program. The next expansion will include an additional course, Biotechnology Industry: Structure and Strategy. The NYU Drug Development Educational Program serves as a framework for other institutions to develop similar, compatible programs to train early stage researchers in this critical area.

## Figures and Tables

**Table 1:** Lecture topics.

Drug Development in a New Era (DDNE)	
Big Data, Supercomputing, Artificial Intelligence Biostatistics and Study Design: New Statistical Approaches and the Application to New Drug Targets Including Metabolic Syndrome/Obesity Biotechnology/Entrepreneurship in Science Brown Adipogenesis as a Target for New Diabetes Drugs Challenges in Drug Discovery for the Treatment of Diabetic Complications Clinical Trials Case Study: Finding Fibromyalgia De-orphanization of GPCRs and Ligand Identification Epigenetic Basis of Metabolic Syndrome and Prenatal Environmental Exposures and Design of Translational Animal Models to Bridge Biomarkers from Mouse to Man and Targeted Drug Selection Prediction Finding the Missing Heritability Wide-Locus GWAS in Pharmacogenomics Fraud and Misconduct Genomic Medicine and Repurposing of Drugs Healthcare in the New Economy Human Subject Protection in Research Infliximab: How a TNF Inhibitor Advanced from Modest Beginnings to Unforeseen Therapeutic Success	Intellectual Property Relating to Clinical Data in Drug Development Modifiers of Gene Expression in Genetic Disease and Measuring Effects on Phenotype Drug Development in Orphan Disease Lysosomal Storage Disorders: Novel Therapeutics Ethics in Clinical Trials, FDA and Conflicts of Interest, PDUFA Moving from Big Data to Better Models of Disease and Drug Response New Diabetes and Obesity Drugs and the FDA, Industry Drug Development Next Generation Data Mining in Pharmacogenomics Patenting Clinical Data Pharmaco-kid-netics: Pediatric Drug Development, an Industry Perspective Pharmacovigilance in Drug Safety, Application of Statistical Data Mining Techniques to Monitor and Predict Drug Safety Phase I/II Developmental Trials Repurposing Failed Drugs to Create Successful Medicines in Women’s Health Drug Discovery and Development: From Target to IND and NDA
Molecular Signaling in the Development and Discovery of Therapeutics (MSDDT)	
Targeting the PI3k-Akt-mTOR Pathway Targeting Metabolic Disease – Primary Efficacy Endpoints for Cardiometabolic Trials Structured-Base Drug/Vaccine Design Targeting HIV/AIDS Startup Biotech: a First Person Perspective on the Risks and Rewards of Starting Your Own Company Signaling Transduction and Signal Management in Pharmacovigilance RNAi for Drug Discovery Receptors and Drug Binding, Pharmacology of Autonomic Nervous System Ras/Cancer RAGE and Diabetic Complications and Therapeutics Approaches Protein Kinases as Targets for Drug Development Principles of G Protein Coupled Receptor signaling Preventative Cardiology Pharmacology of Addiction Personalized Medicine Opioid Receptor Heterodimerization in Analgesia and Addiction Nuclear Hormone Receptors Neuronal Control of Eating Behavior Molecular Signatures Disease Metabolism and Cancer	Medicinal Chemistry Immunotherapy for Tauopathies Glycosaminoglycan Modulation of Signaling Pathways: Implications for Drug Development Drug Distribution, Kinetics, Metabolism, and Cytochrome P450s Drug Addiction: Insights Obtained from Basic Science Research Discovery and Rational Development of an Antagonist to Phosphaturic Tumors Discovery and Rational Development of an FGF23 Hormone Antagonist Diabetic Neuropathy trials and Choice of Endpoints Diabetes and Obesity Computational Drug Discovery Bivalent Approaches to Drug Discovery Approaches and Consideration for Biologic Therapeutic Development – Targeting the FGF Pathway Aldose Reductase and Diabetes Complications Adenosine Receptor Immunomodulators: Discussion between Developer and Industry Partner Macrocyclic Kinase Inhibitors

Abbreviations (in alphabetical order): Acquired Immunodeficiency Syndrome (AIDS), Fibroblast Growth Factor (FGF), Food and Drug Administration (FDA), G-Protein-Coupled Receptor (GPCR), Genome-wide association study (GWAS), Human Immunodeficiency Virus (HIV), Investigational New Drug (IND), Mechanistic Target of Rapamycin (mTOR), New Drug Application (NDA), Pharmacokinetics (PK), Phosphoinositide 3-kinase(PI3k), Prescription Drug User Fee Act (PDUFA), Protein Kinase B (Akt), Receptor for Advanced Glycation End Product (RAGE), RNA interference (RNAi), Tissue Necrosis Factor (TNF).

**Table 2:** Pre-course and post course surveys results. Abbreviation: Standard Deviation (SD)

Drug Development in a New Era (DDNE)						
Question		Pre Course Mean (SD)	Post Course Mean (SD)	Mean Difference	Wilcoxon Signed Rank Test Z-Score	Wilcoxon Signed Rank Test Significance (p)
Knowledge Question		2.57 (0.79)	3.59 (0.43)	1.02	−7.795	<0.001
	2	2.41 (0.72)	3.76 (0.52)	1.35	−7.248	<0.001
	3	2.23 (0.73)	3.71 (0.46)	1.48	−7.886	<0.001
	4	2.61 (0.73)	3.65 (0.52)	1.04	−7.282	<0.001
	5	2.39 (0.69)	3.35 (0.58)	0.96	−6.801	<0.001
	6	1.91 (0.75)	3.22 (0.56)	1.31	−7.496	<0.001
	7	2.03 (0.82)	3.58 (0.5)	1.55	−7.786	<0.001
Total Knowledge Score		16.13 (3.64)	24.69 (2.33)	8.56	−8.099	<0.001
Career Relevance		3.59 (0.57)	3.51 (0.62)	−0.08	−0.480	0.631
Molecular Signaling in the Development and Discovery of Therapeutics (MSDDT)						
Question		Pre Course Mean (SD)	Post Course Mean (SD)	Mean Difference	Wilcoxon Signed Rank Test Z-Score	Wilcoxon Signed Rank Test Significance (p)
Knowledge Question		2.07 (0.82)	3.15 (0.57)	1.08	−5.078	<0.001
	2	2.16 (0.80)	3.35 (0.68)	1.19	−5.116	<0.001
	3	2.27 (0.79)	3.27 (0.57)	1.00	−4.824	<0.001
	4	2.10 (0.82)	3.38 (0.63)	1.28	−5.182	<0.001
	5	2.56 (0.72)	3.46 (0.61)	0.90	−4.885	<0.001
	6	2.31 (0.84)	3.08 (0.57)	0.77	−4.098	<0.001
	7	1.93 (0.75)	3.00 (0.66)	1.07	−5.232	<0.001
	8	2.09 (0.77)	3.23 (0.58)	1.14	−5.114	<0.001
Total Knowledge Score		16.47 (5.44)	25.15 (3.32)	8.68	−5.757	<0.001
Career Relevance		3.64 (0.55)	3.40 (0.85)	−0.24	−1.206	0.228

**Table 3:** Free text responses – Career Relevance Question.

Drug Development in a New Era (DDNE)	
Total Enrollment	139
Number of Pre Course Surveys Completed	127 (91%)*
Number of Students Answered the Career Relevance Free Text Question	113 (89%)**
Categorized Answers	
Number of students indicated interest in career **outside academia** *(keywords: biotech, industry, company, pharma, consulting, biotechnology, pharmaceutical, business)*	40 (35%***)
Number of students indicated interest in working for **research and DDD related careers** *(keywords: research, drug development, clinical trials, basic science)*	66 (58%***)
Molecular Signaling in the Development and Discovery of Therapeutics (MSDDT)	
Total Enrollment	75
Number of Pre Course Surveys Completed	68 (91%*)
Number of Students Answered the Career Relevance Free Text Question	55 (81%**)
Categorized Answers	
Number of students indicated interest in career **outside academia** *(keywords: biotech, industry, company, pharma, consulting, biotechnology, pharmaceutical, business)*	17 (31%***)
Number of students indicated interest in working for **research and DDD related careers** *(keywords: research, drug development, clinical trials, basic science)*	37 (67%***)

* % of students who completed the course;

** % of students who completed the survey;

*** % of students who answered the free response question).

Abbreviations: Chi-square (X^2^), Drug Discovery and Development (DDD), New Drug Application (NDA), Investigational New Drug (IND), Intellectual Property (IP)

**Table 4:** Free response question – What Students Hope to Get Out of the Class (Pre-Course) and What They Got Out of the Class (Post Course).

Drug Development in a New Era (DDNE)		
Total Enrollment	139	
Type of Survey	Pre Course	Post Course
Number of Surveys Completed	127 (91%*)	98 (71%*)
Number of Students Answered the Free Text Question	94 (74%**)	92 (94%**)
Categorized Answers		
Type of Survey	Pre Course	Post Course
Number of students *wanted to learn/learned* the **business and marketing** aspects of DDD *(keywords: property, business, industry, entrepreneur, startup, market, biotech, pharma)* X^2^ p-Value = 0.884	35 (37%***)	31 (34%***)
Number of students *wanted to learn/learned* **regulatory aspects of** DDD *(keywords: regulatory, regulation, approve, approval, FDA, NDA, IND)* X^2^ p-Value = 0.178	14 (15%***)	20 (22%***)
Number of students *wanted to learn/learned* about **intellectual property** *(keywords: IP, protect, intellectual, property, patent)* X^2^ p-Value = 0.034	1 (1%***)	13 (14%***)
Molecular Signaling in the Development and Discovery of Therapeutics (MSDDT)		
Total Enrollment	75	
Type of Survey	Pre Course	Post Course
Number of Surveys Completed	68 (91%*)	52 (69%*)
Number of Students Answered the Free Text Question	63 (93%**)	47 (90%**)
Categorized Answers		
Type of Survey	Pre Course	Post Course
Number of students *wanted to learn/learned* how to **discover a drug** *(key words: inhibit, pathway, target, discovery)* X^2^ p-Value = 1.000	32 (51%***)	24 (51%***)
Number of students *wanted to learn/learned* the **business and marketing** aspects of DDD *(keywords: property, business, industry, entrepreneur, startup, market, biotech, pharma)* X^2^ p-Value = 0.132	9 (14%***)	9 (19%***)
Number of students *wanted to learn/learned* **regulatory aspects of** DDD *(keywords: regulatory, regulation, approve, approval, FDA, NDA, IND)* X^2^ p-Value = 0.317	2 (3%***)	3 (6%***)
Number of students *wanted to learn/learned* about **intellectual property** *(keywords: IP, protect, intellectual, property, patent)* X^2^ p-Value = 0.317	0 (0%***)	1 (2%***)

* % of students who completed the course;

** % of students who completed the survey;

*** % of students who answered the free response question).

Abbreviations: Chi-square (X2), Drug Discovery and Development (DDD), New Drug Application (NDA), Investigational New Drug (IND), Intellectual Property (IP)

**Table 5:** Current fields and disciplines of graduated students who received career development funds.

MD/MSCI Dual Degree Candidates					
Index	Year Received Award	Year of Graduation	Position	Department	Institution
1	2013	2014	Resident	Radiology	NYUSOM, NY, NY
2	2014	2015	Resident	Radiology	Perelman SOM at the University of Pennsylvania, Philadelphia, PA
3	2014	2015	Resident	Pediatrics	Mount Sinai SOM, NY, NY
4	2015	2015	Resident	Dermatology	NYUSOM, NY, NY
5	2016	2017	Resident	Ophthalmology	Johns Hopkins SOM, Baltimore, MD
PhD Candidates					
Index	Year Received Award	Year of Graduation	Position	Department	Institution
6	2016	2016	Post-doctoral fellow	Cardiothoracic surgery	Weill Cornell Medical College, NY, NY
7	2016	2016	Consultant	N/A	ClearView Healthcare Partners (healthcare/consulting) Boston, MA
Masters Degree Candidates					
Index	Year Received Award	Year of Graduation	Position	Department	Institution
8	2013	2014	Senior Clinical Project Coordinator	N/A	QuintilesIMS (healthcare/technology/consulting), Overland Park, KS
9	2016	2017	Clinical Trial Research Coordinator	N/A	NYU Dental School, NY, NY
Post-Doctoral MDs					
Index	Year Received Award	Year of Graduation	Position	Department	Institution
10	2013	2012	Assistant Professor	Internal Medicine/Preventative Medicine/Tropical Medicine	Weill Cornell Medical College, NY, NY
11	2013	2003	Assistant Professor	Neurology	NYUSOM, NY, NY
12	2014	2006	Assistant Professor	Internal Medicine/Nephrology	NYUSOM, NY, NY
13	2015	2008	Assistant Professor	Internal Medicine/Cardiology	NYUSOM, NY, NY
Post-Doctoral PhDs					
Index	Year Received Award	Year of Graduation	Position	Department	Institution
14	2016	2013	Senior Manager	Field Analytics & Operations	Genentech, San Francisco, CA
15	2016	2014	Post-Doctoral PhD	Basic Science and Craniofacial Biology	NYU Dental School, NY, NY
16	2016	1999	Post-Doctoral PhD	Microbiology	NYUSOM, NY, NY
17	2017	2014	Post-Doctoral PhD	Pathology	NYUSOM, NY, NY
18	2017	2013	Post-Doctoral PhD	Endocrinology	NYUSOM, NY, NY
19	2017	2016	Post-Doctoral PhD	Cell Biology	NYUSOM, NY, NY
20	2017	2010	Post-Doctoral PhD	Microbiology	NYUSOM, NY, NY

Abbreviation: School of Medicine (SOM)
